# SARS-CoV-2 with Panton-Valentine leukocidin-producing *Staphylococcus aureus* healthcare-associated pneumonia in the Indian Ocean

**DOI:** 10.1016/j.heliyon.2022.e10422

**Published:** 2022-09-06

**Authors:** Nicolas Allou, Jérome Allyn, Nicolas Traversier, Marie Baron, Renaud Blondé, Céline Dupieux, Nathalie Coolen-Allou, Julien Jabot, Guillaume Miltgen

**Affiliations:** a Réanimation Polyvalente, Centre Hospitalier Universitaire Felix Guyon Allée des Topazes, 97405, Saint Denis, France; b Département d’Informatique Clinique, Centre Hospitalier Universitaire Felix Guyon Allée des Topazes, 97405, Saint Denis, France; c Microbiologie, Centre Hospitalier Universitaire Felix Guyon Allée des Topazes, 97405, Saint Denis, France; d Réanimation Polyvalente, Centre Hospitalier Mamoudzou, Route de l'hôpital, 97600, Mamoudzou, France; e Centre National de Référence des Staphylocoques, Institut des Agents Infectieux, Hospices Civils de Lyon, 69004, Lyon, France; f Pneumologie, Centre Hospitalier Universitaire Felix Guyon Allée des Topazes, 97405, Saint Denis, France; g UMR PIMIT, Processus Infectieux en Milieu Insulaire Tropical, CNRS 9192, INSERM U1187, IRD 249, Université de La Réunion, 97490, Sainte-Clotilde, France; h Regional Antibiotic Therapy Center (CRAtb) of Réunion, France

**Keywords:** Panton-valentine leukocidin, SARS-CoV-2, Mayotte, Reunion Island, *Staphylococcus aureus*

## Abstract

At this time, the literature reports only one case of superinfection with Panton-Valentine leukocidin (PVL)-producing *Staphylococcus aureus* in a patient with severe acute respiratory distress syndrome secondary to coronavirus 2 (SARS-CoV-2) pneumonia. Here we report the first two cases of PVL-producing *S. aureus* healthcare-associated pneumonia in patients hospitalized for SARS-CoV-2 pneumonia in the Indian Ocean region. The two isolated strains of S. aureus were found to belong to the ST152/t355 clone, a known PVL-producing *S. aureus* clone that circulates in Africa and is responsible for infections imported into Europe. Our two cases reinforce the hypothesis that SARS-CoV-2 infection favors the occurrence of PVL-producing *S. aureus* pneumonia. Production of PVL should be searched in patients returning from the Indian Ocean region who present with severe SARS-CoV-2 pneumonia complicated by superinfection with S. aureus even in the case of late onset healthcare-associated pneumonia

*Dear Editor,*

Bacterial superinfections in patients with severe acute respiratory distress syndrome secondary to coronavirus 2 (SARS-CoV-2) pneumonia are relatively rare and are often caused by *Staphylococcus aureus* [1]. At this time, the literature reports only one case of superinfection with Panton-Valentine leukocidin (PVL)-producing *S. aureus* in a patient with SARS-CoV-2 pneumonia[2]. Here we report the first two cases of PVL-producing S. aureus healthcare-associated pneumonia in patients hospitalized for SARS-CoV-2 pneumonia in the Indian Ocean region.

## 1 Case summary # 1

**Figure 1:**
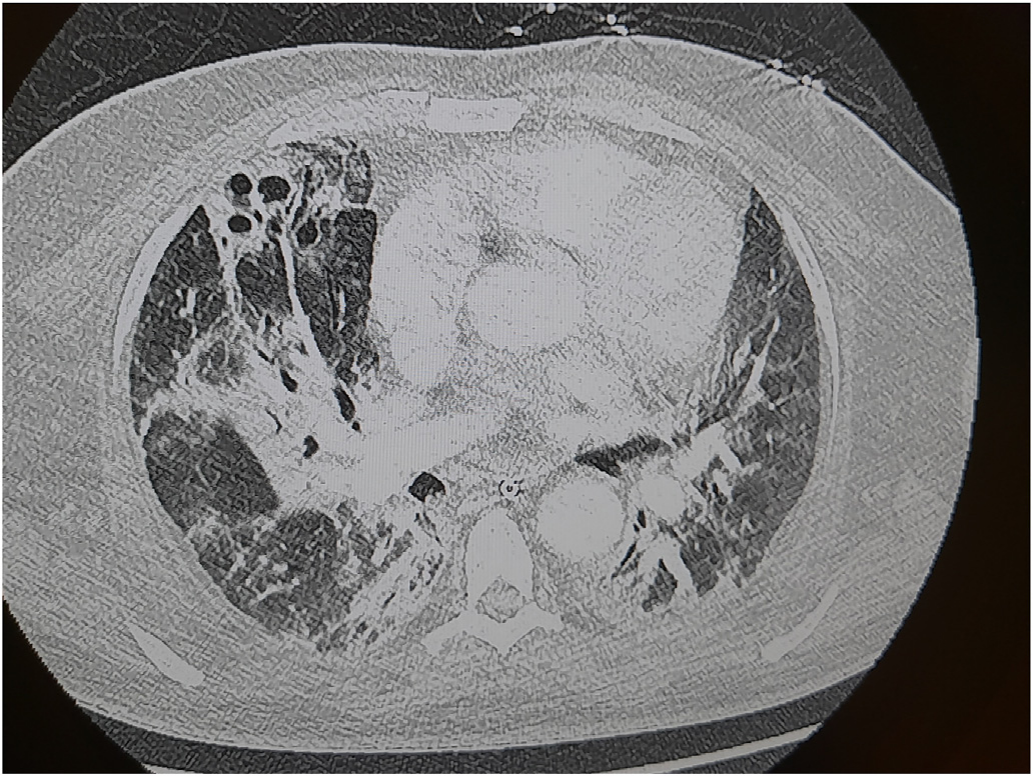
Chest computed tomography-scan performed on day 31 after onset of symptoms showed ground glass opacities and condensations with excavations in right middle lobe.

In February 2021, a 56-year-old obese patient (body mass index (BMI) of 30 kg/m^2^) who was not vaccinated against SARS-CoV-2 consulted the hospital in Mamoudzou, Mayotte, for dyspnea and fever persisting for four days. He was detected positive for SARS-CoV-2 using Polymerase Chain Reaction (PCR) assay. A thoracic computed tomography (CT) scan showed parenchymatous involvement typical of SARS-CoV-2 infection along with bilateral pulmonary embolism. The patient's respiratory status deteriorated 16 days after the onset of symptoms (oxygen saturation of 84% on 15 L/min of oxygen and respiratory rate of 35/min), requiring hospitalization in the intensive care unit (ICU) of Mayotte hospital. On admission to ICU, the patient was treated with invasive mechanical ventilation after failure of high flow nasal cannula therapy. He received dexamethasone, effective anticoagulation, and deworming with albendazole. The evolution was marked by acute respiratory distress syndrome (ARDS) complicated by healthcare-associated pneumonia caused by *Streptococcus pneumoniae* (cytobacteriological examination of the sputum collected on admission was positive for this pathogen). After five days of ICU care, the patient was transferred by plane to Reunion Island to free up beds in Mayotte hospital. On admission to the Reunion Island ICU, microbiological analysis of the tracheal aspirate was negative, procalcitonin was 0.35 ng/mL, and the leukocyte count was 14.9 G/L. On Day 10 of admission, the patient presented with severe ARDS (PaO2/FiO2 of 60 mmHg) due to healthcare-associated bacteremic pneumonia caused by methicillin-susceptible *S. aureus*. The leukocyte count was normal (8.7 G/L). The patient was treated with meropenem and linezolid. A thoracic CT scan showed necrotizing pneumonia (Figure 1). The *S. aureus* strain was positive for PVL by PCR, leading to change antibiotic therapy to oxacillin and clindamycin (for a duration of 15 days). After developing three other nosocomial infections (one healthcare-associated pneumonia caused by *Klebsiella pneumoniae*, one central venous catheter-related bacteremia due to *Pseudomonas aeruginosa*, and one male urinary tract infection with *Escherichia coli*), the patient had a favorable evolution. He was weaned from invasive mechanical ventilation on Day 52 and discharged from the ICU on Day 54.

## 2 Case summary # 2

**Figure 2:**
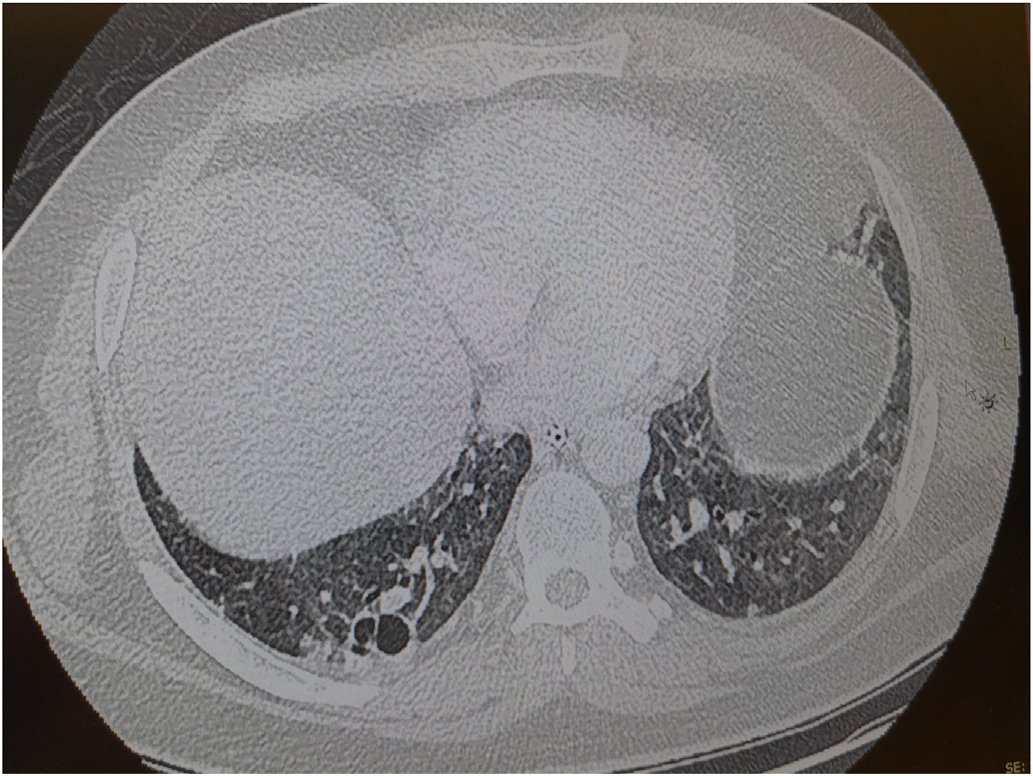
Chest computed tomography-scan performed on day 40 after onset of symptoms showed excavations in right lower lobe with residual ground glass opacities.

In November 2021, a 38-year-old obese patient (BMI of 43 kg/m^2^) with hypothyroidism presented with fever and cough. The patient was not vaccinated against SARS-CoV-2 and had no recent travel history. A PCR performed in a community laboratory was positive for SARS-CoV-2. Five days after the onset of symptoms, the patient was hospitalized in the pneumology department of Reunion Island University Hospital for dyspnea with hypoxemia requiring oxygen at 3 L/min. A thoracic CT scan showed pulmonary involvement typical of SARS-CoV-2 but no pulmonary embolism or abscess (Figure 2). The patient received dexamethasone, deworming with ivermectin, and a tocilizumab injection. Eight days after the onset of symptoms, his respiratory status deteriorated and he was hospitalized in ICU. There was no inflammatory syndrome on admission to ICU (leukocyte count of 6.2 G/L, C-reactive protein of 35.1 mg/L, and procalcitonin of 0.08 ng/mL), but the evolution was rapidly unfavorable. On Day 1 of admission, the patient presented with refractory severe ARDS, which required the use of veno-venous extracorporeal membrane oxygenation. Microbiological analysis of bronchoalveolar lavage fluid was negative. On Day 6, the patient developed sepsis with a leukocyte count of 37.2 G/L. He was treated with piperacillin/tazobactam and linezolid after clusters of Gram-positive cocci were detected in bronchoalveolar lavage fluid and blood cultures. The specimens were then found to be positive for PVL-producing methicillin-susceptible *S. aureus*, and antibiotic treatment was changed to oxacillin and clindamycin for a duration of 14 days days. It was decided not to perform a CT scan because of the difficulty in transporting the patient. The evolution was favorable. On Day 16, the patient was weaned from extracorporeal membrane oxygenation and invasive mechanical ventilation. He was discharged from ICU on Day 36.

The two *S. aureus* strains were identified by matrix-assisted laser desorption ionization time-of-flight mass spectrometry (Microflex, Bruker daltonics, Germany). Antimicrobial susceptibility testing was performed using the disk diffusion and broth microdilution methods according to the 2020 European Committee on Antimicrobial Susceptibility Testing recommendations. The two strains were susceptible to methicillin and to the other antibiotics tested (gentamicin, fluoroquinolones, erythromycin, vancomycin, trimethoprim-sulfamethoxazole, rifampicin) with the exception of penicillin. Detection of PVL genes was performed locally by PCR (RIDA®GENE PVL Real Time PCR kit, R-Biopharm AG, Darmstadt, Germany). The two *S. aureus* isolates detected from blood cultures were then sent for characterization to the French National Reference Centre for Staphylococci (FNRCS) in Lyon. Genotypic analyses for staphylococcal toxins genes using PCR, and typing (*spa*-typing and multi-locus sequence typing, MLST, using whole-genome sequencing) highlighted that both strains harbored the PVL genes and belonged to the sequence type ST152-methicillin-sensitive *S. aureus* treatment and the *spa*-type t355 [3,4].

Here we report the first two cases of PVL-producing *S. aureus* healthcare-associated pneumonia in patients hospitalized for SARS-CoV-2 pneumonia in the Indian Ocean region. An epidemiological study by Vanhecke *et al.* examined all 16 patients who were hospitalized in Reunion Island between 2014 and 2017 for necrotizing pneumonia caused by PVL-producing *S. aureus* (with only one strain being resistant to methicillin) [5]. Of the 16 patients, 14 (87.5%) received intensive care and 6 (37.5%) evolved to death [5]. Leukopenia was observed in only 19% of patients [5], despite the fact that it is described has a classic marker of necrotizing pneumonia caused by PVL-producing *S. aureus* [6]. In line with this finding, the two patients described here did not present with leukopenia. Genetic analysis of the two *S. aureus* strains responsible for pulmonary superinfection in our patients confirmed the presence of PVL and found that the two isolates belonged to the ST152/t355 clone. This PVL-producing *S. aureus* clone has been shown to circulate in Africa (Gulf of Guinea and Southern Africa) and to be responsible for infections imported into Europe [7, 8, 9, 10]. This ST152/t355 clone is also mostly found in PVL-producing methicillin-susceptible *S. aureus* in mainland France (FNRCS data). It has very recently been identified in dairy cow milk in Mozambique, a country involved in the extensive exchange of people and goods (including animals) with Mayotte. Interestingly, two strains harboring the PVL genes but belonging to the *Staphylococcus* argenteus lineage ST2250/2277 have been described in two patients from Mayotte [11].

Our two cases reinforce the hypothesis that SARS-CoV-2 infection could favor the occurrence of *S. aureus* pneumonia by inducing inflammation in the bronchial epithelium and facilitating bacterial adhesion, as described for influenza virus. The presence of PVL may aggravate this pneumonia by preventing an adequate cellular response and promoting the invasive and necrotizing nature of the infection [2]. Although the link between PVL and the severity of healthcare-associated pneumonia due to *S. aureus* has not been clearly demonstrated, and the fact that is difficult to identify the role of this toxin with respect to other *S. aureus* virulence factors in our cases, these two patients presented a notable clinical severity. Production of PVL should therefore be searched in patients returning from the Indian Ocean region who present with severe SARS-CoV-2 pneumonia complicated by superinfection with *S. aureus* even in the case of late onset healthcare-associated pneumonia [12].

## Declarations

### Author contribution statement

All authors listed have significantly contributed to the investigation, development and writing of this article.

### Funding statement

This research did not receive any specific grant from funding agencies in the public, commercial, or not-for-profit sectors.

### Data availability statement

Data included in article/supp. material/referenced in article.

### Declaration of interest’s statement

The authors declare no conflict of interest.

### Additional information

No additional information is available for this paper.
